# Research training program in a Turkish medical school: challenges, barriers and opportunities from the perspectives of the students and faculty members

**DOI:** 10.1186/s12909-020-02454-1

**Published:** 2021-01-04

**Authors:** Zeliha Öcek, Hilal Batı, Ebru Demirel Sezer, Özge Altun Köroğlu, Özlem Yılmaz, Nilüfer Demiral Yılmaz, Aliye Mandıracıoğlu

**Affiliations:** 1 Department of Public Health, Faculty of Medicine, Ege University, Izmir, Turkey; 2 Department of Medical Education, Faculty of Medicine, Ege University, Izmir, Turkey; 3 Department of Medical Biochemistry, Faculty of Medicine, Ege University, 35100 Izmir, Turkey; 4 Division of Neonatology, Department of Paediatrics, Faculty of Medicine, Ege University, Izmir, Turkey; 5 Department of Histology and Embryology, Faculty of Medicine, Ege University, Izmir, Turkey

**Keywords:** Research, Scientific competencies, Medical student, Science education, undergraduate research

## Abstract

**Background:**

Providing medical students with opportunities for research experience is challenging for medical schools in developing countries. The Research Training Program (RTP), which is carried out in Ege University Faculty of Medicine (EUFM) parallel to the core curriculum, aims to improve the scientific competencies of the highly motivated students and to provide them with the opportunity to conduct a research. The purpose of this project is to evaluate RTP through the perspectives of students and faculty members.

**Methods:**

This phenomenological study included two groups; students of RTP and faculty members who contributed to the program. Interviews were conducted with the research group whose selection was determined by maximum variation technique. Interviews with new individuals continued until data saturation was reached. Interpretative data analysis started with close reading of the transcripts and generating a list of codes. Coding by two independently, developing categories and themes were the following steps.

**Results:**

Twenty-one RTP students and 14 faculty members were interviewed. The main motivation for students to participate was the desire to learn how to do research. The introduction course providing the students with the basic competencies needs to be improved in terms of practical activities. It was reported that during the project process students needed intensive guidance especially in finding a research topic and a mentor. The students’ lack of time, deficit of enough mentoring and the fact that conducting a research does not provide a competitive advantage for residency are important obstacles to the completion of the program. The most frequently mentioned achievement of the students is to learn all the stages of the research as well as getting acquainted with critical thinking.

**Conclusions:**

This research showed that it was realistic to implement research programs for highly motivated students in medical schools with conditions like those in EUFM. The solution of mentor shortage emerged in this study is dependent on the adoption of student research as a national policy. Getting acquainted with the interrogative thinking style, conducting research, and making lifelong learning a core value are more important outcomes of research programs than the number of completed projects.

## Background

Furnishing future doctors with scientific competencies is one of the main goals of medical education [1–3]. The significance of integrating research training into education and the benefits of research experiences for medical undergraduates have been well documented in the literature [4–10]. Also, medical students attribute great importance to providing them with the opportunity to conduct a research. They think that this experience can help them develop skills such as critical thinking and decision making as well as providing them the skills for carrying out and presenting a research besides to the advantages for their careers [5, 10–13]. Several studies indicate that exposure to research during undergraduate education strongly affects a career-long interest in research [14–18]. For all these reasons, especially in developing countries, it is essential to encourage medical students to get involved in research experiences [19].

Different approaches are used to provide medical students with scientific competencies and the experience to be involved in research projects. For example, in the U.S., outstanding students are offered the opportunity to pursue a physician-scientist career through a funded MD/PhD program [20]. In a two-year research program implemented in Norway, one of these years is integrated into medical education, while students receive an extra year of education during the other half [21]. Other forms of research engagement are Intercalated Bachelor of Science degrees that are particularly common in the UK and are characterized by research time-out periods between the basic and clinical years of medical school [20]. However, these programs do not comply with the organizational structure and legislation of medical education in many countries. Murdoch-Eaton et al. [5] noted that exposure to research with development of research skills within the curriculum is a more realistic goal and mandatory curricular modules can be used to achieve this goal. There are medical schools in the UK, Ireland and South Africa that follow this strategy [5, 10, 22]. On the other hand, the interest and capacity of all medical students related to research is not at the same level [10]. Therefore, it is also emphasized that the so-called “add-on” approaches that provide research experience can be offered only to the volunteers and / or academically talented students [3, 22–25].

The main outcome of research experiences for medical students is to have an advantage for competitive residency applications [23]. Intense competition for training posts makes research experience an important commodity [8]. Furthermore, in some countries like Germany, it is mandatory for medical students to submit a thesis outlining the results of a research project to graduate with the title “Doctor” [26, 27]. In countries such as Turkey, where scientific competence does not provide a competitive advantage for residency, nor conducting a research project brings any title to medical undergraduates, motivating students to be involved in a research and integrating research training into medical education are very challenging tasks.

In Turkey, students who are in the top 1–3% of achievement levels according to their results of an exam organized by national level are accepted to the medical faculties. Medical education lasts for 6 years and all potential applicants for residency programs need to take a common, national level exam (Medical Specialty Selection Examination, TUS). Those candidates who achieve the highest grades are placed into the specialization programs. In Turkey, where health services are predominantly based on specialty for years, medical students attach priority to their specialty education in their career preferences and include the period of preparation for TUS on their agenda [28–30]. On the other hand, considering that the most successful students in the country are accepted to the medical schools, it is obvious that most of the students have a significant capacity and motivation to conduct research.

### Research training program in Ege University Medical Faculty

Educational activities in the Ege University Faculty of Medicine (EUFM) aimed at providing students with the basic scientific competencies include themes such as introduction to epidemiology, access to information and critical assessment as well as research project planning. Upon observing that this curriculum, which covers all students irrespective of their level of interest, falls short of adequately addressing the needs of students with a more intense scientific curiosity and eagerness to conduct research, a training program has been developed for students who excel regarding their motivation and academic capacity. The objective of this program, called Research Training Program (RTP), was defined as enabling students to meet the philosophy of science and internalize the ethics of science, improving their critical thinking and evidence-based decision making skills, giving them the ability to conduct research in their areas of interest, and designing, conducting, developing and presenting a scientific research as well as publishing it. The RTP was put into practice during the academic year of 2011–2012 with the admission of the first students.

The RTP is announced to the first-year students at the beginning of the spring term of each academic year and 16 students are selected from approximately 50 of 400 students who apply to the program. Students are accepted into the program based on a review of their curriculum vitae and an essay describing their interest and experience in research activities, their performance in the structured interview and their academic performance in the medical school. A commission comprising 12 faculty staff members representing different disciplines executes the RTP. The members of the commission undertake to be the counsellor of at least three students in order to guide them throughout the RTP.

The RTP is composed of two stages. The first stage is the introduction course, which aims to create a scientific infrastructure for the second- and third-year students participating in the program. This course is carried out within the time periods allocated to elective courses in the curriculum. Three quarters of the 208-h course are conducted for eight consecutive weeks during 4 h of a selected day, and the remaining quarter in block form for a full week. The program is carried out in the form of lectures, panels, team-based sessions, visits to research units of the university and assignments. In the second stage of the RTP, which covers the fourth, fifth and sixth grade, students are expected to conduct a research as their primary responsibility under the guidance of a project mentor. The process is initiated with each student determining the field and the project mentor under the guidance of an RTP counsellor. In cases of any need for financial support students apply to the research fund program of the university with their project mentors. During this process, meetings are held, and students present how far they have proceeded through stages. In addition, students have the opportunity to present their projects at the EUFM-RTP Project Festival held every other year. Only students who have completed a project as the first researcher are entitled to receive the RTP Certificate upon their graduation.

The aim of this study is to evaluate the RTP in terms of its different characteristics from the perspectives of students and faculty members who have contributed to the program, and to gain in-depth knowledge of the challenges, strategies and facilitators encountered in the research training and project-making processes of medical students.

## Methods

### Evaluation model and design

Stake’s response evaluation model, which focuses on program activities and processes rather than products, was used in accordance with the purpose of the study. The approach of the responsive model can be applied to summative and formative evaluations. While formative evaluation is useful in monitoring the process and identifying problems, summative evaluation determines the activities, strengths, and shortcomings of the program [31]. Phenomenological approach and interpretative analysis were chosen considering that the study intended to gather data on the phenomenon of RTP from the perspectives of those who had experienced it [32–34]. The central question was whether RTP as a phenomenon was appropriate to provide students with science education and research experience in the current challenges and opportunities. In accordance with the goal of phenomenology, the meaning of RTP experience was described both in terms of what was experienced and how it was experienced [32–34].

### Study population

The study population consisted of two groups. The first group included students of RTP while the second group composed of faculty staff that were members of RTP committee and/or contributed to the program as research mentors or lecturers of the introduction course. Inclusion criterion for students was attending the program for at least one semester, whereas faculty members with at least 1 year of experience in RTP were invited to the study. While interpretative analysis approach deliberately uses small samples of respondents to gather detailed information about their experiences [33]**,** we preferred a maximum variation sampling to obtain a study group diversified according to different characteristics and experiences. For this purpose, the authors decided on the minimum number of the students from each class and from the alumni group. After half of the students were recruited, new participants were invited who would create diversity according to sex and situation in research. Efforts were also made to balance the number of faculty members representing different roles within the RTP (introduction course lecturer, commission member, project mentor). Since only a minority of the faculty members from surgical sciences had assumed a role in RTP, particular attention was paid to the participation of this group. Recruitment continued while new information was emerging and variation within the study group was provided, and it was ceased when saturation was achieved. The study population consisted of 35 participants (14 faculty members, 6 graduates, 15 students).

### Data collection

Data was collected through a semi-structured interview technique. Students were interviewed by a medical education specialist (NDY) who did not have a role in RTP, whereas another medical education specialist (HB) and one public health specialist (ZÖ) who were members of the RTP committee (ZÖ between 2010 and 2016; AHB since 2013) carried out the interviews with faculty members. ZÖ and AHB had read the relevant literature before the interviews began and made a list of key concepts and issues. By taking account of this list, the purpose of the study and the experiences of the authors, four different semi-structured interview forms were developed in a workshop in order to ensure consistent probing across participants; 1) RTP students / graduates 2) Members of the RTP committee 3) Research project mentors 4) Lecturers in the Introduction Course. Faculty members with multiple roles were interviewed using combinations of the forms. Students were asked about their motivation for applying to the program; their assessments on the introduction course; difficulties experienced during the project; impacts of the RTP. The interviews with the RTP committee members focused on the selection process of the students; student-committee relationships; student counselling; execution of the RTP; supporting of projects; impacts of the program; reasons for dropping the program. Questions directed to project mentors were about how they decided to join the program; how the research topic was determined; what the challenges they faced in these research processes were. The lecturers were asked to compare the introduction course with their other educational activities regarding the motivation of the students; learning objectives; etc. The interviewers also maintained research diaries to record their reflections about each interview. All interviews were held face-to face in environments where the speakers were not heard from outside, and the consent of the participants was taken for the audio recording.

### Data analysis

Interpretative analysis was performed in the view of its methodological suitability, particularly the acceptance of the researcher’s influence in data collection and analysis and considering the research team as an integral part of the analysis [33, 34]. Initially, all transcripts were read by three authors (ZÖ, AHB, NDY) one by one after each interview to familiarize with the content. The close reading of the transcripts and highlighting the interesting passages enabled the authors to generate initial open codes and notes describing the striking issues. Once this process has been completed for the whole transcript, the three authors transformed their notes into emerging open and selective codes and then met to create a consensus on a common list of selective codes. After all interviews were coded by two researchers (ZÖ, AHB) independently according to this list, discrepancies in coding were addressed in a meeting with the participation of NDY. While reaching a consensus on coding, the diaries kept during the interviews were taken into the consideration. This was followed by combining the codes and corresponding texts to visualize the grouping of the codes without losing the link with the original data. Eight codes were dropped out since they were not integral to the aim of the study. The final list of the codes consisted of 26 items (Table 1). ZÖ and AHB developed and named categories by looking for connections between the codes and grouping them together according to conceptual similarities. The categories were modified and categorized under themes in a final meeting with the participation of all members of the study team through the discussion on the conceptual framework and each category was illustrated by direct quotes of the participants. The process ended with the validation check for the consistency among final themes, categories, codes, quotes and transcripts conducted by the third author (EDS).

**Table 1:** List of the codes

Code	
Reputability of RTP (reputability)	
Expectations of students (expectations)	
Selection of students (selection)	
Relations among students	
Course program, content, and educational methods (program)	
Students-course lecturer relations	
Placement and timing of RTP in the medical curriculum (placement)	
Seeking for a mentor (seeking mentor)	
Research topic	
Application for ethical approval / financial support (ethical approval)	
Financial support for the projects (financial support)	
University’s research facilities (research facilities)	
Student’s motivation	
Faculty motivation	
Time management	
Unachieved project goals (unachieved goals)	
Student-mentor relations	
Students-commission relations	
Mentor-commission relations	
RTP commission and decision-making processes (Commission)	
Support from the university administration (university support)	
Competencies gained (competencies)	
Quitting RTP (quitting)	
Career choice	
Emotions towards RTP (emotions)	
Certification	

### Quality assurance measures

To ensure the quality assurance of data collection and analysis procedures, credibility, transferability, dependability, and reflexivity criteria were considered [35, 36]. Conducting the interviews through semi-structured forms prepared by a team that has close familiarity with the study phenomenon, carrying out the interviews and coding by the authors who had experiences in qualitative studies, conducting member checks, recruiting participants with different roles and experiences regarding RTP were measures taken for credibility and transferability. To improve the dependability, transcripts coded independently by ZÖ’s and AHB’s were addressed by a third author (NDY) and EDS conducted a final check for validation. Sharing the notes held during the interviews, analysis by two coders from different disciplines, the external perspective provided by NDY, who had no involvement with RTP, discussions of the interdisciplinary study team (medical education, public health, biochemistry, neonatology and histology) to give openness to the new ways of interpreting participant’s perceptions served as a means to provide multiple perspectives and so ensure that the results accurately reflected the data.

## Results

Twenty-one RTP students (six graduates; six last year; eight fifth year; one fourth year) were interviewed. Twelve of the students were female. Of the 14 faculty members interviewed, nine were members of the RTP committee, 10 contributed to the introduction course as lecturers and 10 were project mentors. Three of the faculty members played all the three roles whereas one contributed to the program only as a mentor. Five faculty members were from basic medical sciences, six from internal medical sciences and three from surgical medical sciences.

**Fig. 1:**
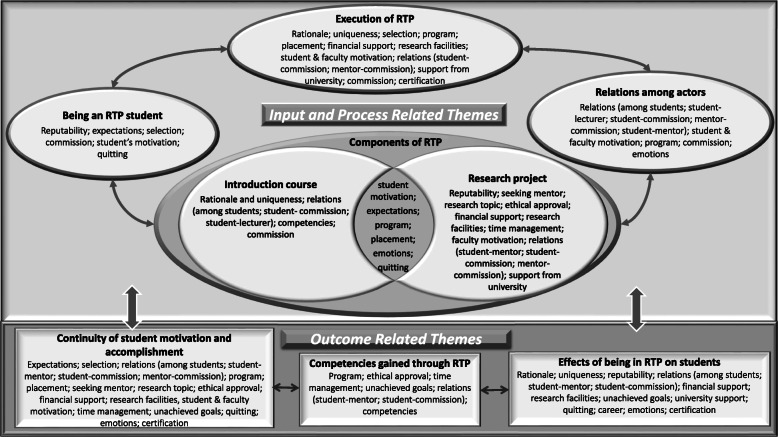
Conceptual framework of the themes and categories emerged in the study

Themes, categories, and selected quotes are presented in Table 2, while Fig. 1 shows the relationship of the themes and categories with one another and the corresponding codes.

**Table 2:** Themes, categories and selected quotes from the participants

Themes	Categories	Quotes
**Being an RTP student**	–	- I applied to be involved in a scientific activity, to have a closer contact with faculty members and observe their work. (fifth grade student, female) - We discussed selection of students for a long time. In the first year, we placed great emphasis on eagerness and did not care much about the academic success. However, we were terribly disappointed. We had selected students who were more social, who presented themselves well, and consequently most of them quit the program. Academic success was in fact an indicator, if a task is given importance, so are other tasks. … We must be able to select those who are prone to research and who are also determined. (CM^1^, ICL^2^, PM^3^, professor, internal medical sciences, male)
**Components of RTP**	Introduction course	- I remember the activities which pushed us to think, e.g. a research was given to be analysed, and we were asked to define the post Phase-2 stages. (sixth grade student, female) - A few years pass until they can apply what they have learnt. I wonder whether they can really practice what I have taught. It is better for them to learn by doing the statistics. This is just a beginning. (ICL^2^, associate professor, internal sciences)
	Research project-1; Finding a mentor and research topic	- They apply to wrong addresses. They get interested in a subject, a faculty member is working on, but that person might reject them probably because of her/his busy schedule or personality and this makes the students feel demotivated. (CM^1^ and ICL^2^, associate professor, female) - I visited many departments. The faculty member in the field I wanted to work told me that he was too busy and directed me to another faculty member, but he could not spare time for me either. Then I changed my topic and conducted my research elsewhere. It was largely a waste of time. (graduate student, female) - I selected my mentor thanks to my RTP consultant. She made an appointment for me with a faculty member in the field I am interested in and we visited my mentor together. Thus, I could start the project in my third grade. (fourth grade student, male) - Students must be interested in a specific area and read a lot. They imagine that the study subject comes to one’s mind out of the blue. (CM^1^ and ICL^2^, professor, basic medical sciences, female) - I was asking myself: How can I create a question without knowing the physiology and the pathology of any subject well? I had a talk with a few faculty members, and they all turned me down as I mentioned above and, in the end, I decided not to commence a project. (sixth grade student, female) - I was aware that asking the right questions and thinking systematically had to do with reading a lot, but I lacked the capacity to do this at that time. (graduate student, female) - My mentor first offered me a few project options. “Which one do you feel drawn to? In the meantime, you can visit all units, see which one is more suitable and select a project accordingly”. Thus, I selected my project in the third grade. (fifth grade student, female)
	Research project-2: Challenges and facilitators encountered during the process	- Getting the ethical approval took 1 year, after the third revision. It might be exhausting. Mentors sometimes assign any subject without thinking whether you can manage it or not. (sixth grade student, male) - We had to buy material for our research. Although I guided the student, I did all the work such as searching for the material. Purchasing took a long time. In the meantime, the Turkish currency depreciated considerably. The appliance we were supposed to use broke down; we could not have it repaired because we didn’t have the money. …. We could not proceed as we had planned, so I had to do the rest myself and explained them how it all worked. (ICL^2^ and PM^3^, professor, surgical sciences, female) - We are still waiting for an answer whether our project will receive financial support or not. I thought the process would be faster and I would play a more active role. I have been exerting great efforts for a long time, but my project has not yet become tangible. (fifth grade student, female) - My mentor invited me to the meetings of his research team so that I could adapt to the subject. I attended every meeting, but this time I started to miss lessons. … Then, I pushed the articles I had to read into the background and drifted apart from the project. However, I was motivated thanks to the support of my mentor. (graduate student, male) - We made a four-year plan in taking account her studentship and my workload. …. We did not need to apply for financial support. Maybe this made our job easier. …. Thanks to her enthusiasm, we passed all phases smoothly. (PM^3^, associate professor, surgical sciences, male)
**Execution of RTP; opportunities and threats**		- There were students who came to us after the lessons and asked questions from a scientific point of view. We felt they had other areas of interest outside of standard classes. The dean’s office at that time also established a commission to provide guidance to these students. (CM^1^, PM^3^, professor, basic sciences, female) - The most important reason for the RTP’s success is that the medical school embraces it and the administration embraces it. So, the students’ interest grows. Even its suspension became an issue for a period. In fact, the administrators except those in that period gave support, but you somehow come to a deadlock. It should be highlighted in faculty demonstrations. In the past, there were students who selected Ege University Medical Faculty just because of the RTP. … The financial situation of our Specialists’ Association is good. When I could not find support from the university, I sent many students abroad thanks to the Association. (CM^1^, ICL^2^, PM^3^, professor, internal medical sciences, male) - We had conducted the project without having financial support; using our own means. But I could not get financial support from the university for the presentation of the project. I was disheartened by this. I took my project to Berlin and South Korea through my own means. (graduate student, female) - I don’t want the RTP commission to be regarded as dreamers either. Financial and moral support should be provided to the RTP. (sixth grade student, male) - Faculty members cannot get involved without feeling the meaning of RTP and the uniqueness of the students. We know them well and personally witnessed their wishes. We do not leave them in the lurch. (CM^1^, ICL^2^, PM^3^, associate professor, basic medical sciences, male)
**Relations among different actors of RTP**		- We encouraged each other instead of being rivals. Two other friends and I developed another project and attended student congresses. It was a motivating atmosphere. (fifth grade student, male) - The RTP commission members were asking us whether we were attending a meeting or not. I felt responsible keeping in mind that “they care for me and trust me”. (sixth grade student, female) - I could not share my problems because I did not have the chance to do so. It could have increased my motivation if we had held more meetings and had seen what our friends were doing and how their solutions were. (fifth grade student, female) - RTP counsellors can help the trouble-facing students to find their way more easily. We sometimes need a hand to push us, so that we can keep going. Even the question “how it is going” helps to motivate one. (Sixth grade student, female) - My mentor never said, “there is nothing to do”. She always said, “You try and research and then we talk about it.” She never discouraged me but showed me the way instead of making the decisions. (graduate student, female)
**Student outcomes of RTP**	Continuity of student motivation and accomplishment of RTP	- I sometimes got tired and questioned if it was the right decision to do this at all. (fourth grade student, male) The project was to be carried out while I was doing internal medicine internship, I was very busy. My mentor often called me. We had several meetings. He used to say, “Read about this subject for 2 weeks”. My English was not so well. I think my priorities have changed. I had the feeling I was lagging behind everyone was studying for “TUS”. (fifth grade student, male) - One’s perception of the world changes as they get older. The ones who wish to do everything end up doing nothing. (CM^1^, ICL^2^ and PM^3^, associate professor, basic medical sciences, male)
	Competencies gained through RTP	- I have learned that it takes 2–3 years to write a 10-page review. (fourth grade student, male) - She worked in every stage of the project and learned all the stages. I could have let her do everything but instead I let her do the job, corrected it and helped her to see the corrections. She visited the labs, observed, and decided on the inclusion criteria. (CM^1^, ICL^2^ and PM^3^, professor, internal sciences, female) - The essence of the university education should be like it is in RTP. It teaches how to think and criticise. (sixth grade student, female)
	Effects of Being in RTP on Students	- I gained an RTP student identity beyond being a medical student. I was being appreciated for being a part of it in different situations. I have been writing to study abroad and have mentioned the fact that I am an RTP student. (fifth grade student, female) - I started my PhD thanks to the RTP. It made me positive that I wanted to be a scientist. (graduate student, male)

*CM* Commission Member, *ICL* Introduction Course Lecturer, *PM* Project Mentor

### Becoming an RTP student

The students indicated that they had participated in the RTP to conduct a research and to learn how a research was carried out. There were those who stated that they had started this process when they were students so that they could become researchers or academicians in the future. Some students said that they had participated with the purpose of questioning the validity of information. More than half of the students reported that they preferred EUFM for the sake of the RTP. On the other hand, the members of the RTP Commission shared that they questioned student selection for a long time and then developed it in due course. Experiences within the process have shown that academic achievement and time management skills are important selection criteria.

### Components of the RTP

#### Introduction course

Students stated that they made better use of practice-based learning activities and suggested that both theoretical and applied activities be planned in a more integrated manner. Faculty members joined this comment but stated that learning objectives remained at the introductory level and applied activities could not be achieved adequately as the students were in the early years of medical education. Faculty members believe that education which is divided into specific periods cannot provide integrity. Students also criticized the dense schedule where the lessons were squeezed into one afternoon of a week and disclosed that they broke off from the process within a week, and the “project writing” course in the form of a one-week module enabled them to focus uninterruptedly.

#### Research project

More than half of the students conveyed their negative experiences with faculty members to whom they or their friends applied to do projects together. Five mentors stated that the students approached them after the session organized by the RTP Commission during which research fields and voluntary mentors were introduced, though a student could not achieve any results in this way. Seven students were able to reach a mentor through the RTP consultants. However, an RTP counsellor’s mediation may not always be enough. Two RTP consultants also undertook the project mentoring of the student as a last resort. A faculty member suggested that students and faculty members from the pool of mentors should be matched up in accordance with their interests.

Students had difficulties in finding the project topics and their interaction with the academic faculty staff can sometimes demotivate them. The faculty members also explained that the students were not able to generate ideas or directed towards unrealistic issues such as mind mapping. The statements that have been conveyed show that students can generate questions only under the guidance of mentors. On the other hand, guidance sometimes results in selection of the topic by the mentor. The reasons for this are the impracticability of the conditions for the topics that the students have in mind and the necessity to find, within a short period of time, a topic that can be applied to the student who has encountered various setbacks. Mentors expressed also that it was not easy to persuade students to conduct applicable studies. Suggestions were made to facilitate topic selection such as students’ developing a research idea after gaining experience by joining the team of an ongoing project; generating a list of possible topics that students could pick from. A faculty member suggested that students join a research team instead of conducting a project under their own responsibility, while some participants stated that the students selecting this option would only cause the students to act as assistants performing only limited tasks due to hierarchical relations.

The students stated that preparing an application for ethical approval or financial support was much more difficult than they had expected. Students also said that they could lose their motivation and that they were able to get over these difficulties only with the support of their mentors. Most of the participating students expressed that they could not spare time for their research due to the intensity of medical education and anxieties related to TUS in the following years. Faculty members argued that starting research in an early period of the students’ education might reduce these problems, in which case the scope of the research would be limited because of their knowledge level. Concerning their research, students emphasized the importance of an early start and time management. It was also expressed that allocating time periods in the curriculum to the RTP after the third grade would ensure the completion of the projects. Remarks made by one counsellor and two students provided clues to an effective process in which the mentor would help the student adapt to the research environment, have him/her conduct a preliminary study, create a work schedule, give short-term assignments, hold regular meetings and maintain constant communication. It is frequently recommended that students first participate in a study as observers and then carry out their own studies after gaining some experience. On the other hand, two graduates who worked this way stated that all they did was limited to the care of experimental animals and that they could not participate in the research planning processes.

### Execution of RTP

The four founding faculty members of the commission stated that the starting point of the formation of the RTP was the requirement of the students’ eagerness based on their capacity to conduct scientific research; they had discussed many different program options during the planning stage; and a program was eventually shaped according to the conditions of Turkey.

Members of the commission pointed out that the support of the faculty staff and the university administration was critical for the success of the RTP and expressed that the RTP should be made more special and popular. Since there was not enough support for students to participate in congresses, the RTP Commission had to seek the necessary support from external organizations. Moreover, fluctuations in foreign currency caused a serious deficit between the financial support and the expenses made for the projects.

The RTP Commission and students found it difficult to find faculty members who would accept to be mentors in clinical sciences. Commission members often had to undertake the role of mentors to bridge this gap. All participants believed that the RTP should be possessed by the whole faculty in order to increase the number of mentors. Other suggestions included motivational tools such as certificates of appreciation, academic promotion criteria, etc.

### Relations among different actors of RTP

While most students stated that they had positive and supportive relationships with their fellow RTP students, their relationships with the students in other classes remained limited. One student explained the reason why there was no competition between them as “refraining from touching one another”. The importance of a close relationship between the commission and the students was emphasized. Seven students stated that they did not have any individual communication with the commission as they did not need to while one student described the relationship between the commission and the students as “hierarchical”. Two faculty members shared their distress stemming from the inability of the commission to notice some students who reached the point of quitting the RTP. The importance of scientific and social activities that would bring together the commission and the students was mentioned, and it was suggested that the frequency of these activities be increased with the purpose of strengthening the weakened bonds following the introduction course.

Members of RTP Commission emphasized the importance of providing individual counselling to students. Sixteen students stated that they received support from the RTP counsellors at every stage. However, four counsellors expressed that some students did not establish communication with them despite all their efforts. It was stated that problems were largely solved thanks to a system initiated recently requiring that the counsellor and the student hold regular meetings.

All the students who demonstrated progress in their projects referred to a positive relationship with their mentors. The positive features emphasized included more easily accessible mentors who motivate, and enable students to conduct their projects by instructing them. On the other hand, there were also students who lost their motivation and quit the program due to the poor mentoring.

### Student outcomes of the RTP

#### Continuity of student motivation and accomplishment of the RTP

Three students in the research group quitted their projects. Despite some differences, the statements reflected that the students’ got demotivated in time; that they lost heart when confronted with difficulties such as bureaucratic procedures, and the interest of their mentors and students’ perseverance was decisive in the continuation of their motivation. Statements revealed that the students who were late in determining a topic could not start or carry with a project due to their responsibilities in the clinical period and the anxiety related to TUS. According to students and commission members, the reasons for quitting the program included the mistake in student selection; failure to understand the sense of straightforwardness in the project process; poor time management; unrealistic topics; problems with the mentor; lack of support from the counsellor; and the weakening connection between the commission and students after the introduction course. According to two faculty members, quitting voluntary programs was natural. Four faculty members and one graduate student referred to the negative impact of the fact that the certificates awarded to those who completed the program did not earn them a title like a master’s degree. Commission members expressed that support should be given to those who lost their motivation for reasons beyond their control such as not getting efficient mentoring.

#### Competencies acquired through the program

The most frequently mentioned achievement of the students is to learn all the stages of the research. The more active role the student played, the more outcomes they attained. One of each three students published and/or presented his/her research in a congress and described it as “the realization of the dreams”. Five participants stated that the project process displayed the obstacles to be encountered in real life and the strategies to be followed. Another frequently emphasized achievement was getting acquainted with critical thinking. Four students emphasized that this aspect of the RTP was an example of university education in the real sense. Six participants added that the RTP improved also communication skills and reduced hierarchy. Some students expressed that they had learned from their mistakes related to time management.

#### Effects of being in the RTP on students and emotional experiences

The RTP was defined as an original program of EUFM and 11 students stated that taking part in the RTP made them special. Taking part in the RTP opened other doors such the opportunity for working at research centres abroad. The statements made by five participants, reflected that the RTP shaped students’ career preferences. Having been able to progress through the project was quite decisive in terms of feelings towards the RTP. These particular students conveyed their emotions toward the RTP process using expressions such as feeling more knowledgeable, experienced and luckier than other students as well as being privileged because they can be in a scientific environment, thinking that they had a real university education, and feeling a sense of belonging to the university.

## Discussion

This study provided important clues on how to enable highly motivated students to improve their scientific competencies and gain research experiences in countries where there is no national policy encouraging medical undergraduates to do research. The RTP experience in EUFM revealed the challenges encountered in research training in medical schools with very high number of students and daily routine heavy workload of faculty members and indicated strategies to be followed for an effective process.

Studies conducted in different countries have demonstrated that students want to receive research training so as to acquire research skills, learn to think critically, make scientific publications, shape their career plans and establish interaction with faculty members [3, 6–8, 37, 38]. Similar motivational reasons were reported both in the present study and in another study conducted 4 years ago which evaluated the RTP [39], but career planning remained behind the other reasons. This was expected since making research applications prior to graduation in Turkey does not provide a direct benefit. The coherence between the rationale behind the establishment of the RTP and the students’ motivational reasons has revealed the necessity of research programs that contain higher objectives than the curriculum. Study results have indicated that medical students vary with respect to their interests in research and a considerable portion of the students prefer special programs for the ones who are strongly interested in research in addition to scientific core curriculum. Thus, these results support the volunteer-based program approach [10, 26]. Moreover, difficulties related to mentoring and funding revealed by this study also reflects that only voluntary approaches are applicable for similar faculties. However, it should be emphasized that the existence of a program specific to a single group cannot be an alternative to a medical curriculum based on basic principles of scientific research as well as evidence.

Murdoch-Eaton et al. [5] suggested that scientific competencies of students should be developed in the early stages of their medical education. Similarly, in EUFM, all students get acquainted with science-related topics in the first year and the RTP starts at the beginning of the second year. The results of the research [3, 40] indicating that the duration of the programs that last several months, such as summer schools, is not sufficient for the completion of projects; that programs expanding over years yield more research show that it is logical to spread the RTP over 5 years. On the other hand, our findings have reflected that continuity is critical during these 5 years. The course in the first 2 years furnishes the students with basic competencies in research; however, students cannot integrate with this application because they have not built up conceptual knowledge and have not started research projects yet. The statements of the participants reflect that a more spiral curriculum can enable some learning objectives to be transferred to other years, thereby minimizing the disconnection to be experienced after the introduction course. However, time-protected activities should be allocated to the RTP in the last 3 years. In this manner, the problem of not having sufficient time for research especially during the later years of education, as both reported by RTP students and frequently encountered in the literature [9, 11, 19, 37, 41, 42].

The statements of all participants show that students required intensive support from RTP counsellors during the process of finding a project topic and a mentor. Students whose interests were determined by their counsellors and aided to meet with key people and visit related clinics and laboratories were able to progress further. For this reason, their counsellors should be in close contact with students even if they do not actively demand counselling themselves. The newly initiated regular meeting system has greatly reduced the dependence of counsellor-student relationships on personal traits. It is stated that not only the counsellors but also the RTP Commission itself can play a significant role in determining project topics and prepare a list of suitable topics for the students who need help. Another suggestion which consisted of listing the names of volunteer project advisors with research proposals was successfully executed at the University of Texas [23]. Our findings indicated that mentors in such programs should be informed by the coordinators about students’ qualifications, points of support, feasible research topics, and the boundary between mentoring and making decisions on behalf of the student.

The fact that students found formulating a research question and hypothesis as well as preparing ethics committee and project application files more difficult than they had expected could be a natural consequence of the learning process. The same difficulties were reported in other studies and some students described such difficulties as a valuable learning experience [5, 42]. In any case, this learning process can only be successfully completed with the support of the mentors [43, 44]. The mentor should direct the student to a project topic that may be attractive and feasible, discuss the literature together, structure the project proposal preparation processes, and supervise time management. Moreover, the key qualities of a good mentor in this study emerged as being easily accessible, answering questions in a short time, engaging in the learning process with the student and acting in a motivational manner. As stated in the literature, in order that students could play an active role rather than just “passive” mechanical assistantship or “passive” pure data collection, the mentor should make sure that the student understands all stages of the research and masters the essence of the research, which calls for experienced faculty members who can strike a balance between their roles as researchers and educators and do not consider the time spent on students as wasted [5, 19, 45]. Nevertheless, not getting efficient mentoring was among the problems most frequently reported by the students [3, 5, 19, 23, 40]. The fact that most the students participated in our study were able to find a mentor and had a positive communication may have led to a more positive picture than the existing one. Acknowledgements of the participating students concerning the problems encountered by their friends, rather than themselves, also support the possibility mentioned above.

It is a common problem for many medical faculties, especially those in developing countries, to provide students with opportunities for research experience during pre-graduate medical education and to convince faculty members to become mentors [6, 9, 11, 19, 40, 42, 46–48]. Our findings have revealed that the novel idea of performing research with students coupled with the intensity of daily routine workload limits the number of volunteers. Participants believe that the RTP should be embraced by the whole faculty to increase the number of mentors. Universities where faculty members are encouraged to support student research in line with institutional policies are good examples in this context [10]. Recommendations put forward in this study in order to increase the number of mentors included explaining the advantages of working with students to the faculty members; introducing factors that encourage mentoring such as academic promotion criteria; and assigning non-medical faculty members as advisors. In consequence, as suggested by Tamariza et al. [47], universities wishing to support student research need to create a pool of mentors.

Some of the participants recommended that students gain experience by joining another project team before starting their own work. However, except for a few successful examples in the scope of which this proposal was made, there were instances where students were only used as a labour force without having a chance to develop their own research skills. Therefore, this recommendation should be implemented only after the student’s tasks are clearly defined and the RTP counsellor is properly informed. The members of the RTP Commission expressed that the suggestion that the students join an existing research team instead of conducting a project under their sole responsibility could lead to a similar result. The study conducted by Murdoch-Eaton et al. also supported this caution. When 475 projects that students engaged in were evaluated in terms of skill acquisition, the research methods remained at the 31% level and it was found that students were assigned tasks such as finding patients and entering data [5].

The motivation of the students decreases dramatically during the project stage, and hence some of them quit the program because of some difficulties they encounter. The decrease in the dropout rate observed in recent years was explained with the better structured process and with the inclusion of academic achievement as a selection criterion. The study conducted by Salgueira et al. [48] has also demonstrated that academic achievement is related to student engagement in scientific activities. On the other hand, as declared by participants, it would be unrealistic for all students to complete such programs. Problems encountered by RTP students such as not being able to devote time to research alongside their education and changing their priorities have been reported in other programs as well [9, 11, 19, 23, 37, 41, 42, 48, 49]. Moreover, unlike many countries [3, 5, 6, 17], completing the RTP does not give an advantage in competition for the specialty education or even creates a disadvantage in terms of time spent for exam preparation. Lack of institutional incentives such as obtaining a valid certificate or a title upon completion discourages also the students from conducting research [11, 50]. What should be done here is to support students who come to the point of quitting RTP not because of lack of external motivation but because they do not receive enough mentoring or financial support.

It has been reported that conducting a research enables medical students to understand research methods and furnishes them with skills such as generating research questions, planning and executing research, analysing data, authoring articles, making scientific presentation [3, 5, 6, 10, 23, 47, 49]. Students who successfully completed the RTP also learned their research process through experience. However, as is the case with RTP, the learning outcomes of courses in research are not limited to the realization of a project. Research is a learning process and its outcomes are not just output-oriented [43, 44]. Consequently, even those who could not complete their project have undoubtedly gained considerable benefits from this process. Research programs such as RTP offer students the opportunity to be in an environment where interrogative thinking is the core value. Furthermore, the RTP has also influenced career preferences of the students. Publications stating that research experience acquired as student results in a research-oriented career choice in the future, and that research and lifelong learning become a behavioural pattern for the individual also supports this potential impact of the RTP (6, 49). In addition to all these, the RTP has also improved students’ communication and time management skills as other research programs [3, 5, 10]. On the other hand, the statements of the participants revealed that more emphasis should be placed on time management skills, and mastering teamwork skills, which is reported as another outcome of research training [10, 23].

Program coordinators assume a critical role in maintaining continuity of research programs, sustaining a supervisory relationship between the students and the faculty, and creating a positive pedagogical atmosphere [3, 42]. Our findings demonstrate the importance of forming an RTP Commission from stable members who are competent in research training, have communication and empathy skills, act as role models for students, and are determined to devote time to the program. The fact that members represent different disciplines paves the way for a broader perspective. Students suggested that the frequency of scientific and social meetings be increased to strengthen the positive atmosphere. On the other hand, the support of the university administrations is imperative for executors of the research programs [23]. Suggestions made at this point included ensuring that RTP becomes more special and popular, promoting project mentoring, finding resources for the projects, and encouraging the students to participate in scientific congresses.

The students interviewed in this research feel privileged due to the scientific environments they can be a part of. However, these positive emotions belong to students who have been able to progress in their projects. Chang [3] also reported that students who successfully completed their research had positive perceptions regarding the process. Lagging in the project stage weakens students’ emotions and sense of belonging towards the program. Reluctance of the students, who have not completed their projects, to attend the meetings may be stemming from their negative feelings towards the program. In addition to this source of participation bias, another limitation of the study is that students possibly refrain from expressing their negative emotions. Moreover, as Chang [3] stated, the outcomes reported by the students rely on their self-assessments. Such outcomes need to be evaluated basing on objective criteria. The fact that the study is based on the experience in a single faculty is the limitation of the generalizability of our results.

This research showed that it was realistic to implement research programs designed for highly motivated and talented students as well as educational activities that provide basic scientific skills to all students in medical schools with conditions like those in EUFM. Our results also pointed to the need to plan these programs over years in a spiral manner by preserving their integrity. Students need the intensive and structured support of program executives and mentors. Listing the names of volunteer mentors together with prospective research suggestions can facilitate the process of finding mentors and research topics for students. The starting point for the solution to the mentor shortage is the adoption of student research as a policy at a national and university level. Besides, medical schools can develop specific strategies in accordance with their conditions. Program coordinators should guide the mentors throughout the whole process; closely monitor all student processes; and provide timely support to students experiencing problems. On the other hand, coordinators need to be supported by university administrations in order to perform these functions. The outcomes of research programs like the RTP should not be evaluated only as output-oriented processes aimed at carrying out and publishing a project. Instead, getting acquainted with the interrogative and systematic thinking style, conducting research, and making lifelong learning a core value should be considered as the most important program outcomes.

## Data Availability

The datasets used and/or analysed during the current study are available from the corresponding author upon reasonable request.

## Abbreviations

EUFM: Ege University Faculty of Medicine
RTP: Research Training Program
TUS: Medical Specialty Selection Examination (Tıpta Uzmanlık Sınavı)
CM: Commission Member
ICL: Introduction Course Lecturer
PM: Project Mentor
